# Supplementary material for: GOFlowLLM—curating miRNA literature with large language models and flowcharts
Source: Bioinformatics. 2026 Jan 6;42(1):btaf683. doi: 10.1093/bioinformatics/btaf683 (PMC12790822; doi:10.1093/bioinformatics/btaf683)
Supplement: btaf683_Supplementary_Data [file btaf683_supplementary_data.pdf]

Example LLM curation report

Below is an example of the curation report generated from GoFlowLLM's output, as seen by the curators assessing the system. Each section between +++ lines relates to a node in the flowchart: a single yes/no question the LLM is asked. The question is filled from the flowchart definition, then everything following that up until the "Evidence: " line is generated by the LLM as part of its reasoning.

The text after "Evidence:" up to "Decision:" is a guaranteed substring of the paper that provides evidence to support the assertion the LLM makes. The "Decision:" line is simply a restatement of the constrained decision made by the LLM, where in some cases the reasoning has been curtailed by limiting the tokens generated.

This example shows the LLM being able to reason about the presence of assays, the targets of those assays and in this case the outcome of the assay. This example was rated correct for all evaluations - the reasoning, evidence and selected GO terms are all correct when re-assessed by a curator, and is an example of a paper where the previous annotation has been deepened from a less specific term to a more specific term by looking at the outcome of the qRT-PCR assay.

The final proposed annotation below is generated programmatically given the terminal node reached in the flowchart and the LLM-identified targets. It simply encodes all the GO terms that are present in the flowchart diagram at that node (see main text Figure 1)

++

We are curating the paper PMC4737258, into one of the following three GO terms:
GO:0035195 - miRNA-mediated post-transcriptional gene silencing - A post-transcriptional gene silencing pathway in which regulatory microRNAs (miRNAs) elicit silencing of specific target genes. miRNAs are endogenous 21-24 nucleotide small RNAs processed from stem-loop RNA precursors (pre-miRNAs). Once incorporated into a RNA-induced silencing complex (RISC), miRNAs can downregulate protein production by either of two posttranscriptional mechanisms: endonucleolytic cleavage of the RNA (often mRNA) or mRNA translational repression, usually accompanied by poly-A tail shortening and subsequent degradation of the mRNA. miRNAs are present in all the animals and in plants, whereas siRNAs are present in lower animals and in plants. PMID:14744438 PMID:15066275 PMID:15066283 PMID:23209154 PMID:23985560 PMID:28379604

GO:0035278 - miRNA-mediated gene silencing by inhibition of translation - An RNA interference pathway in which microRNAs (miRNAs) block the translation of target mRNAs into proteins. Once incorporated into a RNA-induced silencing complex (RISC), a miRNA will typically mediate repression of translation if the miRNA imperfectly base-pairs with the 3' untranslated regions of target mRNAs. PMID:14744438 PMID:15196554

GO:0035279 - miRNA-mediated gene silencing by mRNA destabilization - An RNA interference pathway in which microRNAs (miRNAs) direct the cleavage of target mRNAs. Once incorporated into a RNA-induced silencing complex (RISC), a miRNA base pairing with near-perfect complementarity to the target mRNA will typically direct targeted endonucleolytic cleavage of the mRNA. Many plant miRNAs downregulate gene expression through this mechanism. PMID:14744438 PMID:15196554 PMID:21118121 PMID:23209154

++

Question: Does the paper have a functional interaction between the miRNA and an mRNA, determined by a reporter assay? Acceptable assays include: Luciferase reporter assays where a luciferase reporter gene is fused to the 3'UTR of the target mRNA; and CRISPR/CAS9 deletion of the miRNA response element with subsequent measurement of protein levels.

1. The question asks if the paper shows a functional interaction between miR-199b and an mRNA via a reporter assay (e.g., luciferase with 3'UTR or CRISPR deletion).
2. Scanning the text for miR-199b reporter assays:
   - The "Luciferase Reporter Assay" section states: "cells were transfected with the JAG1 3′UTR Lenti-reporter-Luc Vector (ABM), and the Pre-199b, LNA-199b and controls." Luciferase activity was measured to assess miR-199b effects.
   - This directly uses a 3'UTR luciferase reporter for JAG1, testing miR-199b's interaction with its mRNA target.
3. No mention of CRISPR/Cas9 or miRNA response element deletions related to miR-199b.
4. The luciferase assay with 3'UTR fusion meets the criteria for a functional interaction test.

Conclusion: Yes, the paper uses a luciferase reporter assay with the JAG1 3'UTR to test miR-199b's interaction with its mRNA target.

Evidence: cells were transfected with the luciferase plasmids under the control of the promoter of the VEGF receptor (Addgene [plasmid 21307] generated by Mammoto et al.) 23, the JAG1 3′UTR Lenti-reporter-Luc Vector (ABM), and the Pre-199b, LNA-199b and controls.

Decision: Yes

++

Question: Does the paper include an assay for the miRNA effect on the target gene mRNA expression? This must be a direct assay of the levels of mRNA for the gene identified as a target of the miRNA, not inferred from a binding assay or other measurement. If other things are measured with qRT-PCR, such as to confirm the expression of the miRNA, you should ignore them.

1. The question asks if the paper directly measured the target gene's mRNA levels in response to miR-199b manipulation, using an assay like qRT-PCR.
2. Scanning the text for miR-199b target mRNA measurements:

- In the "MiR-199b Targets the Notch Ligand Jagged1" section: "Pre-199b suppressed the expression of JAG1 and Notch 1 while the LNA-199b induced the expression of both JAG1 and Notch 1 (Fig. 3A–3D)." Figure 3A mentions mRNA levels for JAG1 and Notch1.
- The "Luciferase Reporter Assay" section mentions a 3'UTR reporter for JAG1, but this is a binding assay, not direct mRNA quantification.
- The "Results" section states: "luciferase assays revealed that the 3′UTR of the JAG1 is a direct target of Pre-199b during EC differentiation from iPS cells (Fig. 3E)." This confirms miR-199b binds to JAG1's 3'UTR.
- However, the text explicitly mentions qRT-PCR for JAG1 mRNA levels in response to miR-199b overexpression/knockdown (Fig. 3A shows mRNA levels).

3. The question specifies a direct assay of the target gene's mRNA (not just protein or reporter activity). The JAG1 mRNA levels were measured via qRT-PCR in Figure 3A, as stated in the text.
4. Other qRT-PCR results (e.g., for miR-199b itself or EC markers like CD144) are irrelevant per the question's restriction.

Conclusion: Yes, the paper includes qRT-PCR assays measuring JAG1 mRNA levels in response to miR-199b manipulation.

Evidence: Pre-199b suppressed the expression of JAG1 and Notch 1 while the LNA-199b induced the expression of both JAG1 and Notch 1 (Fig. 3A–3D). In addition, luciferase assays revealed that the 3′UTR of the JAG1 is a direct target of Pre-199b during EC differentiation from iPS cells (Fig. 3E).

Decision: Yes

++

Question: After transfecting the miRNA and observing decreased protein levels, did the authors observe decreased mRNA levels of the miRNA target gene? This must be an observed change in the mRNA levels of the target gene using a technique such as qRT-PCR, not an inference from other measurements. Make sure the qRT-PCR is used to measure the target gene mRNA level, not just the miRNA level.

1. The question asks if decreased target gene mRNA levels were observed via qRT-PCR after miR-199b overexpression (leading to lower protein).
2. Focus on miR-199b's target genes. From prior analysis, JAG1 is a target.
3. In the "MiR-199b Targets the Notch Ligand Jagged1" section:
- "Pre-199b suppressed the expression of JAG1 and Notch 1 while the LNA-199b induced the expression of both JAG1 and Notch 1 (Fig. 3A–3D)."
- Figure 3A specifically mentions mRNA levels for JAG1 and Notch1.
- The text states that "luciferase assays revealed that the 3′UTR of the JAG1 is a direct target of Pre-199b," but this is a binding assay, not mRNA quantification.
- The suppressed expression of JAG1 mRNA by Pre-199b (miR-199b overexpression) is directly stated, implying qRT-PCR was used (as per earlier mention of qRT-PCR for mRNA measurements).

4. The question requires that decreased protein AND decreased mRNA (via qRT-PCR) are both observed after miR-199b overexpression.

- The text says Pre-199b "suppressed the expression of JAG1" in both mRNA (Fig. 3A) and protein (Fig. 3C).
5. Thus, the authors observed decreased JAG1 mRNA (via qRT-PCR) and protein when miR-199b was overexpressed.

Conclusion: Yes, the authors observed decreased JAG1 mRNA levels via qRT-PCR when miR-199b was overexpressed, alongside reduced protein levels.

Evidence: Pre‐199b suppressed the expression of JAG1 and Notch 1 while the LNA‐199b induced the expression of both JAG1 and Notch 1 (Fig. 3A–3D).

Decision: Yes

++

Question: What is the gene name of the mRNA targeted by this miRNA?

1. The question asks for the gene name(s) of the mRNA targeted by miR-199b in the paper, from the provided list.
2. From previous analysis, the paper explicitly states that miR-199b targets JAG1:
   - "luciferase assays revealed that the 3′UTR of the JAG1 is a direct target of Pre-199b during EC differentiation from iPS cells (Fig. 3E)."
   - "Pre-199b suppressed the expression of JAG1 and Notch 1 while the LNA-199b induced the expression of both JAG1 and Notch 1 (Fig. 3A–3D)."
3. JAG1 is in the provided list as "JAG1" and "Jagged1". The paper uses "JAG1" (e.g., "JAG1 3′UTR Lenti-reporter-Luc Vector").
4. Other genes like STAT3 or VEGF are mentioned in the context of miR-199b's regulatory effects but are not direct targets demonstrated in the paper. For example:
   - STAT3's expression is linked to miR-199b via transcriptional activation, but the paper does not show miR-199b directly targets its mRNA.
   - VEGF's secretion is regulated, but the target is JAG1, which indirectly affects VEGF via the Notch pathway.
5. The only explicitly demonstrated target from the list is JAG1/Jagged1.

MiRNA's target protein name(s) as per the paper: JAG1 (also referred to as Jagged1 in the text).

The final answer is: JAG1 (or Jagged1, but the paper uses JAG1)
Evidence: luciferase assays revealed that the 3′UTR of the JAG1 is a direct target of Pre‐199b during EC differentiation from iPS cells (Fig. 3E).

Target: JAG1

++

Final proposed annotation:

[
  {
    "BP": {
      "involved_in": "GO:0035279"
    },
    "MF": {
      "enables": "GO:1903231",
      "part_of": "GO:0035279"
    },
    "has_input": "JAG1",
    "reference": "PMC4737258",
    "rna_name": "miR-199b"
  }
]
